# Responsive Guest Encapsulation of Dynamic Conjugated Microporous Polymers

**DOI:** 10.1038/srep28784

**Published:** 2016-06-30

**Authors:** Lai Xu, Youyong Li

**Affiliations:** 1 Institute of Functional Nano & Soft Materials (FUNSOM), Jiangsu Key Laboratory for Carbon-Based Functional Materials & Devices, Soochow University, 199 Ren’ai Road, Suzhou, Jiangsu, 215123, PR China

## Abstract

The host-guest complexes of conjugated microporous polymers encapsulating C_60_ and dye molecules have been investigated systematically. The orientation of guest molecules inside the cavities, have different terms: inside the open cavities of the polymer, or inside the cavities formed by packing different polymers. The host backbone shows responsive dynamic behavior in order to accommodate the size and shape of incoming guest molecule or guest aggregates. Simulations show that the host-guest binding of conjugated polymers is stronger than that of non-conjugated polymers. This detailed study could provide a clear picture for the host-guest interaction for dynamic conjugated microporous polymers. The mechanism obtained could guide designing new conjugated microporous polymers.

The guest encapsulation behavior of conjugated microporous organic polymers as a host have attracted a lot of attention by researchers all over the world^1^,^2^,^3^,^4^,^5^. These polymers have wide applications such as luminescence^6^,^7^, sensing^8^,^9^, and photocatalysis^10^. Now scientists could make it possible to achieve easy and fast encapsulation of guest molecules under ambient conditions. This is because of their flexible backbones. They have amorphous three-dimensional organic framework, which provides noncovalent confinement for guest molecules. Therefore, it is easy to tune host-guest composition without changing the polymer structure itself^1^.

Rao *et al.* reported guest-responsive reversible swelling in a dynamic microporous polymer network poly-tetraphenyl pyrene (Py-PP). It encapsulates C_60,_ dye molecules red-emitting 4-(dicyanomethylene)-2-methyl-6-(4-dimethylaminostyryl)-4H-pyran (DMDP) and Nile red (NR) dyes at room temperature^2^,^3^. Although there are extensive experimental studies in host-guest interaction for conjugated organic frameworks, there is no computational investigation on the detailed and dynamic picture of guest encapsulation inside the porous structures. Here, we have investigated the orientation of guest molecules inside host frameworks and responsive dynamic behaviour of host backbone computationally for the first time. Our simulation results match experiments and predict new insights to the detailed mechanism of guest encapsulation. Based on the mechanism, we proposed several ways to design new materials. We also performed control simulations for host-guest interactions of non-conjugated system polydivinylbenzene (PDVB).

## Results

### Construction of Building Units

**Figure 1:**
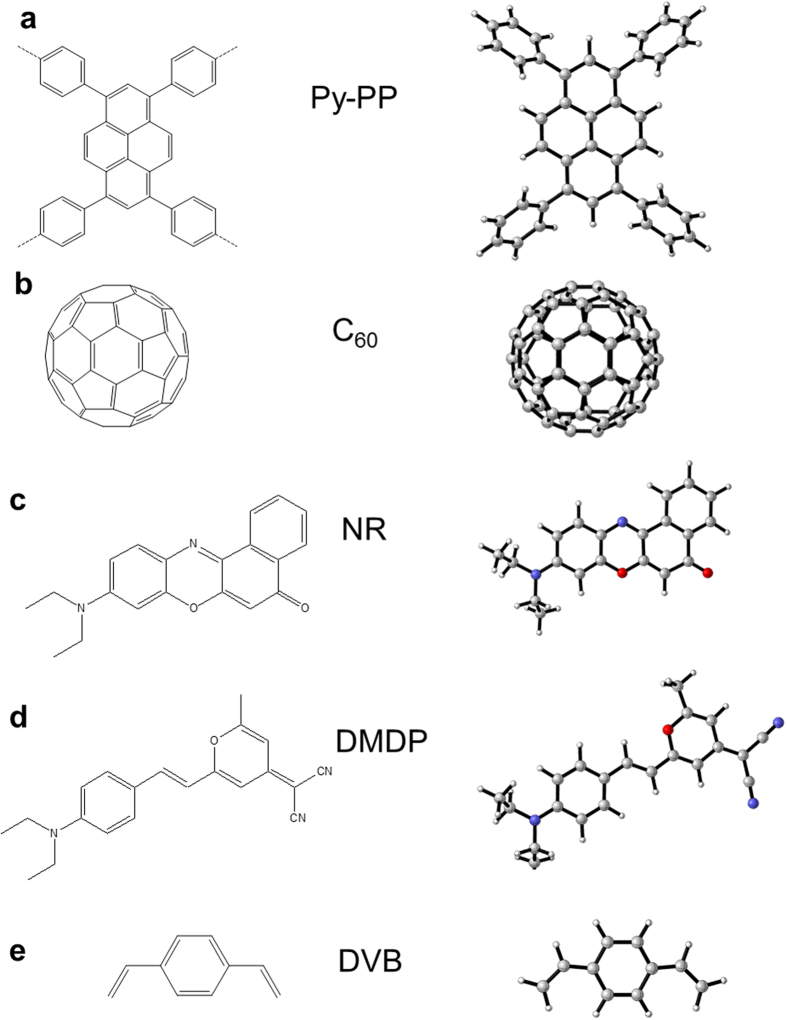
Host polymer monomer of PyPP, monomer of PDVB and guest molecules C_60_, NR, DMDP, and DVB. Structures on the right are optimized structure by B3LYP/6-31G(d).

We chose PyPP as host materials to encapsulate C_60_, dye molecule DMDP, and dye molecule NR. We first obtained stable structure of building units of the host-guest system. Here, we used density functional theory to optimize the geometry of building units. Figure 1 shows monomer of PyPP, as well as guest molecules C_60_, NR, DMDP, and monomer divinylbenzene (DVB) for control simulations. The right column shows the optimized geometry of PyPP monomer, C_60_, NR, DMDP and DVB, by using DFT method B3LYP/6-31G(d)^11^,^12^,^13^ in Gaussian09^14^. The detailed Gaussian reference and structure information could be found in the supplementary information.

**Figure 2:**
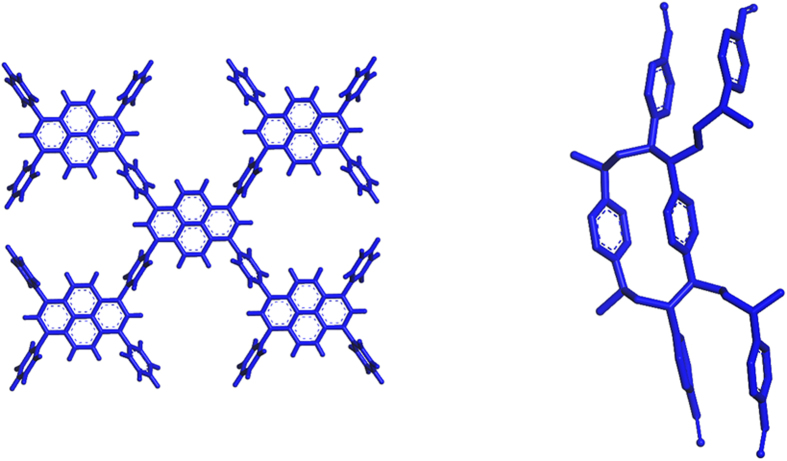
Optimized structure of oligomer of PyPP and PDVB.

Based on the PyPP monomer, we constructed PyPP oligomers and PDVB oligomer for next-step construction of host-guest complex. Here, we used Dreiding^15^ force field to optimize the oligomers. Figure 2 shows the optimized structure of PyPP oligomers and PDVB oligomers by Dreiding^15^ force field in Forcite module of Material Studio 7.0^16^. From the optimized structures, we noticed that the PyPP oligomer is extended structure, with linking phenylene orthogonal to the main extended structure plane. The unique conjugated structure of the backbone will generate interesting phenomena when it encapsulates a guest molecule or guest aggregates. The aromatic interaction could occur near the linker phenylene, or near the pyrene plane. Therefore, this structure provides various possible interaction sites when guests are encapsulated. For PDVB oligomer, since crosslinking behavior of divinylbenzene due to its double bonds, we constructed the oligomer with several monomers crosslinked together. From the structure after geometry optimization, we could see that it is three dimensional structure without conjugated structure. Thus PDVB oligomer is constructed to provide control simulation results for conjugated polymer system PyPP.

### Single Guest Molecule Encapsulation

**Figure 3:**
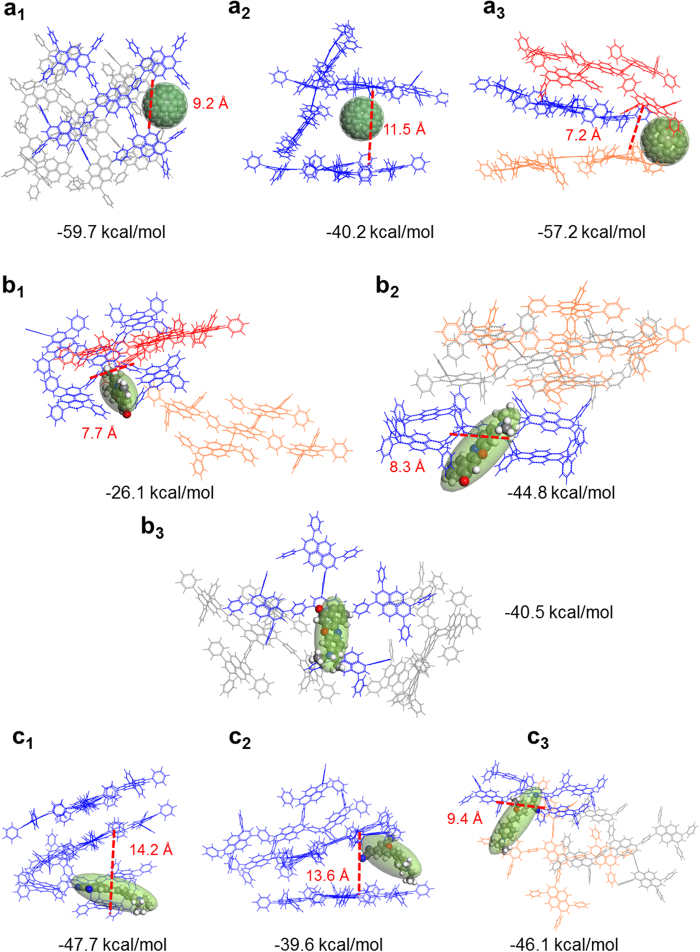
(**a**) Representative orientations of C_60_ molecule encapsulated inside PyPP host. (**b**) Representative orientations of NR molecule encapsulated inside PyPP host. (**c**) Representative orientations of DMDP molecule encapsulated inside PyPP host.

In order to study the molecule orientation of single guest molecule inside host framework, three PyPP oligomers with one guest molecule C_60_, NR, and DMDP were loaded respectively within Amorphous Cell module in Materials Studio 7.0^16^. Fifteen stable configurations were generated from Configurational Bias Monte Carlo method^17^,^18^,^19^ with periodic boundary conditions, and three representative configurations were selected for PyPP-C_60_, PyPP-NR, and PyPP-DMDP complex respectively. Then geometry optimization was performed in Forcite module in Materials Studio 7.0^16^ by Dreiding^15^ force field. We also calculated binding energy of these complex structures. Binding energy was calculated based on the formula ∆E = E(complex) − E(host) − E(guest). The three representative configurations with calculated binding energies of PyPP-C_60_ complex, PyPP-NR complex, and PyPP-DMDP complex are shown in Fig. 3. We chose large system (3 packed oligomers) as our host to calculate binding energies here. The reason is that previous study showed that the calculation of CO_2_ binding energy based on small molecule fragment is not accurate, because it does not include the entire framework^20^.

Firstly, the encapsulation of C_60_ was investigated. Figure 3a shows C_60_ encapsulated in the host framework. It indicates that C_60_ could stay inside the open pore of PyPP (a_1_), or stays within the cavities formed from packed oligomers (a_2_ and a_3_). The binding energies were computed to be −59.7, −40.2 and −57.2 kcal/mol respectively for a_1_, a_2_, and a_3_ orientations. Since the binding energy is calculated from the equation ∆E = E(complex) − E(host) − E(guest), the negative value of binding energy means that the system is stabilized after guest binding. The host guest interaction is mainly noncovalent π-π interaction.

Secondly, we studied NR encapsulation in PyPP framework. Figure 3b shows various orientations of NR residing in the host framework. In Fig. 3b1,b2, NR molecule resides in the open pore and is orthogonal to the pyrene plane, with different orientations. In Fig. 3b3, NR molecule is parallel to the pyrene plane. The binding energies for NR with host were computed to be −26.1, −44.8 and −40.5 kcal/mol for b_1_, b_2_, and b_3_ orientations respectively. The negative value of binding energies indicates that binding process of NR is energetically favorable. The main host-guest interaction is π-π stacking with different orientations.

Lastly, the host-guest interaction between PyPP and DMDP was explored. Figure 3c shows three different configurations for this interaction. Figure 3c1 indicates that DMDP molecule is parallel to the pyrene plane. In Fig. 3c2, DMDP resides in the cavity formed from packing oligomers. Figure 3c3 shows that DMDP is inside the open pore, on the edge of the oligomer. The binding energies for DMDP were calculated to be −47.7 kcal/mol, −39.6 kcal/mol, and −46.1 kcal/mol for c_1_, c_2_, and c_3_ respectively. The negative value also indicates that binding of DMDP molecule to the host framework is energetically favorable. The main host-guest interaction is dipolar interaction between DMDP and aromatic rings in the host framework^3^.

### Control Simulations for Host-Guest Interaction of PDVB

**Figure 4:**
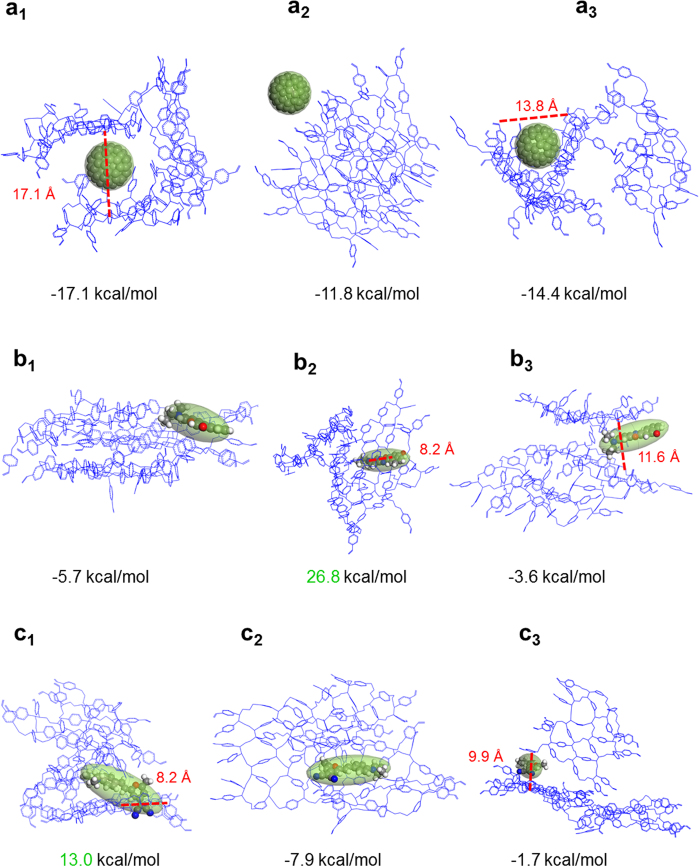
(**a**) Representative orientations of C_60_ molecule encapsulated inside PDVB host. (**b**) Representative orientations of NR molecule encapsulated inside PDVB host. (**c**) Representative orientations of DMDP molecule encapsulated inside PDVB host.

Figure 4 shows the host-guest interaction for PDVB oligomers. We packed three PDVB oligomers and one guest molecule C_60_, NR, and DMDP respectively. We used the same computational methodologies as used for PyPP oligomers. Interestingly, the binding energy of PDVB is much smaller than that of PyPP, indicating that the host guest interaction for PDVB is weaker. For C_60_ molecule, it mainly stays inside the cavity formed by packing multiple oligomers, such as a_1_ and a_3_. C_60_ could also stay on top of the framework such as a_2_. Next, host-guest interaction for NR and DMDP were also explored. The simulations also show that NR and DMDP reside in the cavity formed from packing oligomers such as b_3_ and c_3_. They could also be parallel to the surface of the framework, such as b_1_ and c_2_. Interestingly, we also found configurations where NR or DMDP is inside the closed pore of the PDVB framework. However, the binding is not energetically favorable in this case, since the binding energies were calculated to be positive (see green values in b_2_ and c_1_).

### Guest Aggregates Encapsulation and Responsive Pore Accommodation

Rao *et al.* mentioned that when the loading of guest molecules is increased, they detected the existence of aggregate guest molecules^3^. Here, we investigated the detailed guest aggregation picture computationally. We used Amorphous Cell module to construct two PyPP oligomers and 15 guest molecules C_60_, NR, and DMDP. Similarly, multiple configurations were obtained by Configurational bias Monte Carlo method^17^,^18^,^19^ with periodic boundary conditions. Dreiding^15^ force field in Forcite module was used to optimize the representative configuration geometries.

**Figure 5:**
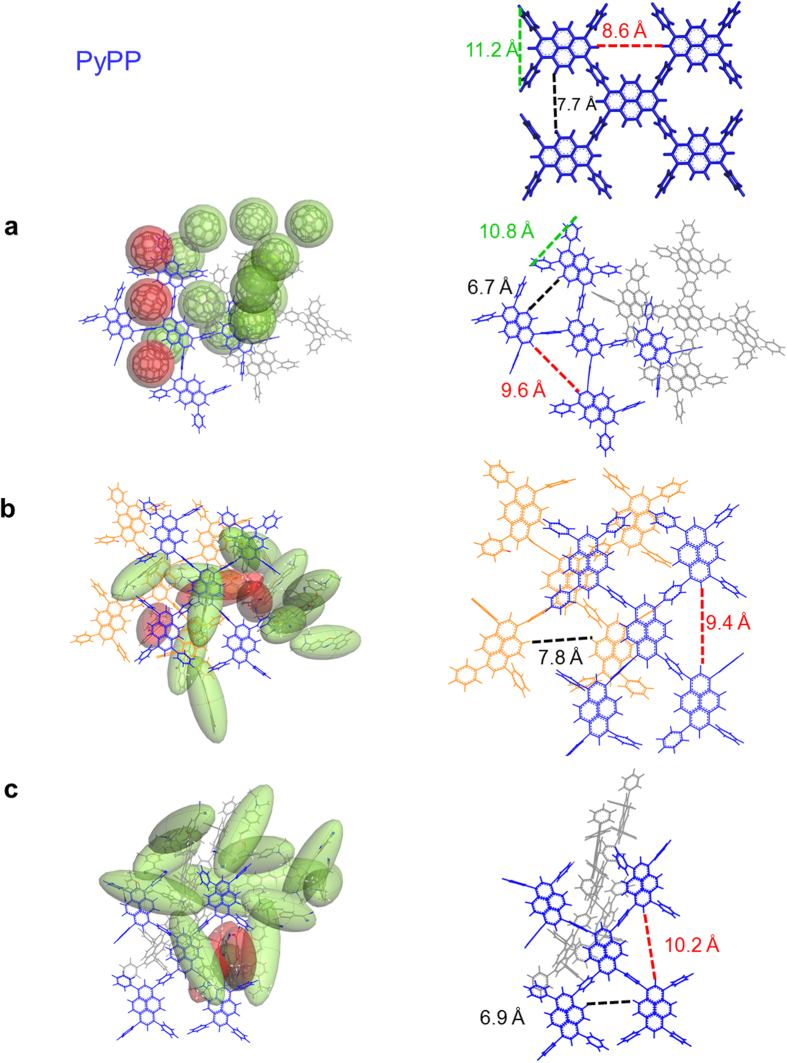
Optimized structure of C_60_, NR and DMDP aggregates encapsulated inside packed PyPP oligomers.

Figure 5 shows the representative encapsulation results for C_60_, NR, and DMDP guest aggregates. The right column of Fig. 5 shows the comparison between the pore size of PyPP oligomer without guest encapsulation, and PyPP oligomer with guest encapsulation. First of all, fullerene aggregates encapsulation was explored. Figure 5a indicates that C_60_ aggregates assembled on top of pyrene arrays. This matches previous experiment well^2^,^21^, which showed that the porous PyPP framework provides an assembled array of fullerenes. After near-surface spots are occupied, more C_60_ molecules will reside layer-by-layer with respect to the pyrene plane. From the comparison between PyPP oligomer pore and C_60_ guest-encapsulated pores in red, the width of three open pores changes. One increases from 8.6 Å to 9.6 Å, the second decreases from 7.7 Å to 6.7 Å. The third open pore decreases from 11.2 Å to 10.8 Å. This is because the aromatic arms of the open pore will adjust its size in order to enhance the host-guest π-π interaction with C_60_ molecule.

Then the host-guest interaction between PyPP and NR aggregates was investigated. Figure 5b shows NR aggregates inside the cavities. NR molecules reside in the open pore of the oligomer. By comparing PyPP oligomer pore and three pores in red, the width of open pore on the left becomes larger from 7.7 Å to 7.8 Å. For the open pore encapsulating two NR molecules on the right, the width of the pore increases from 8.6 Å to 9.4 Å. This clearly demonstrates the dynamic behavior of oligomer backbones. The pore expands in order to accommodate two large-sized NR molecules.

Lastly the encapsulation of DMDP aggregates was studied. Figure 5c shows DMDP aggregates inside the cavities. The width of the open pore has increases from 8.6 Å to 10.2 Å. Since the aggregates with multiple DMDP molecules needs larger space, the open pore will provide larger cavity to encapsulate the aggregates. The open pore will also adjust its shape to accommodate to the shape of aggregates. At the same time, because of the expansion of the open pore, the connected pore shrinks (width decreases from 7.7 Å to 6.9 Å). This indicates the dynamic behavior of the PyPP backbone. In terms of aggregate fashion of PyPP-C_60_, PyPP-NR, and PyPP-DMDP complexes, it follows π-π stacking and C-H-π interaction.

## Discussion

When the guest orientation changes within the host framework, the host-guest interaction will also change. Figure 3a indicates different binding energies with different orientations for the PyPP-C_60_ system. When C_60_ molecule is inside the open pore of the system, the host-guest interaction is strong, such as 3a_1_. When C_60_ molecule is inside the cavity formed from packing oligomers, depending on the packing density, the host-guest interaction varies. If the oligomers are densely packed, the host-guest binding is strong, such as 3a_3_. However, if cavity formed from packing is large, the host-guest interaction will be weaker, such as 3a_2_.

NR molecule interacts with host framework with various orientations. NR could be parallel with the framework, or inside the open pore. When the NR molecule is inside the open pore, depending on the orientation of NR, the binding energy varies. If the interaction occurs between only a portion of NR molecule and the host framework, binding is weak, such as 3b_1_. However, if the contact area between NR and host framework is large, leading to larger host-guest interaction, such as 3b_2_. When NR molecule is parallel with the host plane, such as Fig. 3b3, the contact area between host and guest is large, therefore the host-guest interaction is strong.

In terms of the interaction between DMDP and PyPP framework, it has several different types as well. In Fig. 3c1, DMDP molecule plane is parallel to the host plane. In this case, the interaction between DMDP and the host plane is thorough, thus the binding energy is also large. On the other hand, in Fig. 3c2, DMDP resides in the cavity formed by packing oligomers, and DMDP molecule is not parallel to the host plane. The binding is not that strong in this case. When DMDP molecule is inside the open pore of the host framework (such as 3c_3_), the binding energy is also large, indicating that the interaction between DMDP and edge of the polymer is very strong.

From the trend of Fig. 3 discussed above, we get the conclusion that the interaction between guest molecule and host framework could be attributed to several reasons. First is the compactness of the host framework. Second is the contact area between host framework and guest molecule, depending on the distance between host and guest molecule, and the orientation of guest molecule. Third is that interaction between guest molecule and open pore of the host framework is very strong.

Besides conjugated polymer PyPP, we also performed control simulations of host guest interaction for PDVB, which is non-conjugated polymer. As shown in Fig. 4, the host-guest binding of non-conjugated polymer is not as strong as conjugated polymer. We proposed that the difference depends on whether it is conjugated polymer. For non-conjugated polymer PDVB, the oligomer is formed by crosslinking and the structure is three dimensional. The multiple phenylene rings are not at the same plane and separate from each other (see Fig. 2). When guest interacts with the non-conjugated polymer, the interaction is between aromatic rings of guest and separate phenylene rings in PDVB. On the other hand, for the conjugated PyPP system, the π system is conjugated and extended structure (see Fig. 2), therefore the host-guest interaction is between guest aromatic groups with a large conjugated and extended π system. Thus the interaction between the host and guest molecule is greatly enhanced. We also found that the binding process of guest molecule inside the closed pore of PDVB is not energetically favorable. It takes a high energy barrier (26.8 kcal/mol for NR and 13.0 kcal/mol for DMDP), for the guest molecule to go inside the closed pore of PDVB. Instead, guest molecule tends to stay in the cavity formed by packing PDVB oligomers.

For the pore size change after the guest aggregate as shown in Fig. 5, we find that the dynamic behavior of flexible host backbone of conjugated polymer is the key to accommodate the inclusion of guest aggregates. Firstly, if the guest is relatively small, the pore will shrink to catch the guest. For example, in Fig. 5a, the top two C_60_ molecule was inside the open pore, and the open pore will close the arm in order to catch C_60_. The open pore shrinks in order to catch guest molecule and keep it locked within the pore. Secondly, if the size of the guest aggregates is larger than the pore size, the pore will expand in order to encapsulate the aggregates, such as the right pore of Fig. 5b, and the right pore in Fig. 5c. Lastly, when the pore expands (right pore in Fig. 5c), the neighboring pore (left pore in Fig. 5c) will shrink accordingly. This is also due to the flexibility of the backbone.

**Figure 6:**
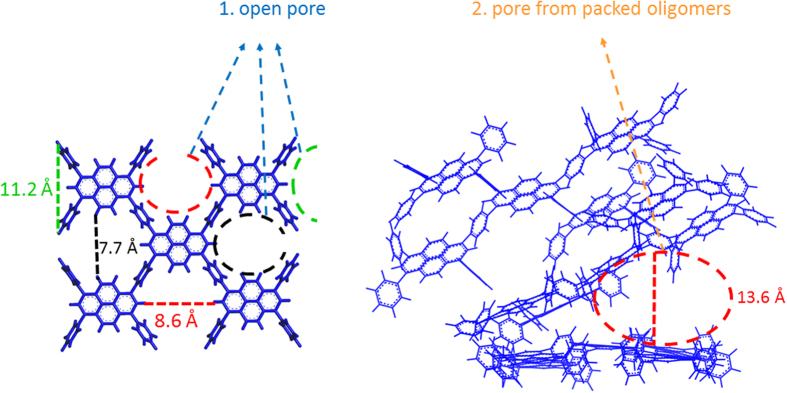
Two different types of pores within PyPP host framework.

From the simulations, we discovered that there are two types of cavities within the PyPP host framework. Figure 6 shows these two pore types. The first type is the internal open pore formed between pyrene and phenylene groups. The second type is cavity formed from packing different PyPP oligomers. For the open pore, there are also three types: the pore labeled in red, black and green respectively. The red pore has diameter of 8.6 Å, black pore has diameter of 7.7 Å, and green pore has diameter of 11.2 Å. For the cavity formed from packing oligomers, it could also be divided into two types, one is that guest molecule is parallel to the framework surface, the other is that guest molecule is deep inside the cavity from packing oligomers.

In conclusion, we explored guest encapsulation for conjugated microporous polymers by theoretical calculations for the first time, discovered different orientations of guest molecules inside the host framework, and confirmed the responsive dynamic behaviour of the polymer backbone by simulations. We also performed control simulations for non-conjugated polymers. The host guest interaction for conjugated polymers is stronger than that of non-conjugated system. This work provides detailed picture of guest encapsulation and could be helpful guidance for future design of dynamic porous materials. For example, future design could focus on new conjugated microporous polymers with pore size appropriate to the size of guest. We found two types of pore and get new insights of open pore, and pore formed from packing oligomers. In order to increase the host-guest interaction, we could adjust the monomer size, or adjust the compactness of the backbone, and ultimately increase the interaction area between the host and guest molecule or guest aggregates.

## Methods

The structure of PyPP monomer, C_60_, NR, DMDP and PDVB monomer, were optimized by using DFT method B3LYP/6-31G(d)^11^,^12^,^13^ in Gaussian09^14^. The PyPP oligomers was geometry optimized by Dreiding^15^ force field in Forcite module of Material Studio 7.0^16^.

The guest encapsulation inside PyPP host framework, was studied by Amorphous Cell module of Material Studio 7.0^16^. PyPP oligomers with guest molecule or guest aggregates of C_60_, NR, and DMDP were loaded respectively. Multiple stable configurations were generated from Configurational Bias Monte Carlo method^17^,^18^,^19^ with periodic boundary conditions, and representative configurations were selected for PyPP-C_60_, PyPP-NR, and PyPP-DMDP complex respectively. Then geometry optimization was performed in Forcite module in Materials Studio 7.0^16^ by Dreiding^15^ force field.

The host guest interaction between PDVB host framework and guest molecule C_60_, NR, and DMDP, was also studied by the same methods mentioned above.

## Additional Information

**How to cite this article**: Xu, L. and Li, Y. Responsive Guest Encapsulation of Dynamic Conjugated Microporous Polymers. *Sci. Rep.* **6**, 28784; doi: 10.1038/srep28784 (2016).

## Supplementary Material

Supplementary Information
