# Supplementary material for: Responsive Guest Encapsulation of Dynamic Conjugated Microporous Polymers
Source: Sci Rep. 2016 Jun 30;6:28784. doi: 10.1038/srep28784 (PMC4928063; doi:10.1038/srep28784)
Supplement: Supplementary Information [file srep28784-s1.pdf]

Supplementary Information

Responsive Guest Encapsulation of Dynamic Conjugated Microporous Polymers

Lai Xu,* and Youyong Li

Institute of Functional Nano & Soft Materials (FUNSOM), Jiangsu Key Laboratory for Carbon-Based Functional Materials & Devices, Soochow University, 199 Ren'ai Road, Suzhou, 215123, Jiangsu, PR China

Full Reference of 14

Gaussian 09, Revision A.1: Frisch, M. J.; Trucks, G. W.; Schlegel, H. B.; Scuseria, G. E.; Robb, M. A.; Cheeseman, J. R.; Scalmani, G.; Barone, V.; Mennucci, B.; Petersson, G. A.; Nakatsuji, H.; Caricato, M.; Li, X.; Hratchian, H. P.; Izmaylov, A. F.; Bloino, J.; Zheng, G.; Sonnenberg, J. L.; Hada, M.; Ehara, M.; Toyota, K.; Fukuda, R.; Hasegawa, J.; Ishida, M.; Nakajima, T.; Honda, Y.; Kitao, O.; Nakai, H.; Vreven, T.; Montgomery, J. A., Jr.; Peralta, J. E.; Ogliaro, F.; Bearpark, M.; Heyd, J. J.; Brothers, E.; Kudin, K. N.; Staroverov, V. N.; Kobayashi, R.; Normand, J.; Raghavachari, K.; Rendell, A.; Burant, J. C.; Iyengar, S. S.; Tomasi, J.; Cossi, M.; Rega, N.; Millam, J. M.; Klene, M.; Knox, J. E.; Cross, J. B.; Bakken, V.; Adamo, C.; Jaramillo, J.; Gomperts, R.; Stratmann, R. E.; Yazyev, O.; Austin, A. J.; Cammi, R.; Pomelli, C.; Ochterski, J. W.; Martin, R. L.; Morokuma, K.; Zakrzewski, V. G.; Voth, G. A.; Salvador, P.; Dannenberg, J. J.; Dapprich, S.; Daniels, A. D.; Ö.Farkas; Foresman, J. B.; Ortiz, J. V.; Cioslowski, J.; Fox, D. J.Gaussian, Inc., Wallingford, CT, 2009.

B3LYP/6-31G(d) optimized geometries of stationary points

Py-PP monomer E(RB3LYP) = -1539.64193158 a.u.

C	-0.72045	-0.0001	-0.04549
C	0.720415	-6.8E-05	-0.04555
C	1.43772	1.244117	-0.05383
C	0.681951	2.463666	-0.13346
C	-0.68196	2.4637	-0.1327
C	-1.43768	1.244165	-0.05282
C	-1.43769	-1.2443	-0.05441
C	-2.85896	-1.23137	-0.04434
C	-3.52694	-6.6E-05	-0.05141
C	-2.85895	1.231253	-0.04211
C	2.859019	1.231129	-0.04364
C	3.527057	-0.00015	-0.05084
C	2.858948	-1.23143	-0.04268
C	1.437697	-1.24427	-0.05375
C	0.681956	-2.46376	-0.13447
C	-0.68195	-2.46376	-0.13495
C	-5.32078	4.774949	0.096261
C	-4.31967	4.581191	1.055475
C	-3.5076	3.443956	1.007401

C	-3.6788	2.476833	-0.00359
C	-4.69444	2.682637	-0.95816
C	-5.50578	3.820342	-0.9104
C	-5.31928	-4.77643	0.085404
C	-4.31938	-4.58377	1.046033
C	-3.50764	-3.44619	1.000647
C	-3.67894	-2.47695	-0.00832
C	-4.69412	-2.6811	-0.96382
C	-5.50411	-3.81993	-0.91942
C	5.318661	4.776915	0.084583
C	4.318884	4.584501	1.045456
C	3.507703	3.446452	1.001046
C	3.678782	2.476888	-0.00766
C	4.693687	2.680956	-0.96338
C	5.503766	3.819684	-0.91959
C	5.321345	-4.77479	0.096864
C	5.508466	-3.81876	-0.90803
C	4.697002	-2.68119	-0.95615
C	3.678842	-2.47699	-0.00389
C	3.505336	-3.44569	1.005293
C	4.317918	-4.58254	1.053903
H	1.217282	3.400667	-0.22243
H	-1.21738	3.400697	-0.22099
H	-4.61121	-0.00011	-0.01059
H	4.611319	-0.00013	-0.00942
H	1.217323	-3.40069	-0.22366
H	-1.21727	-3.40071	-0.22437
H	-4.17621	5.310744	1.846609
H	-2.74819	3.290302	1.767289
H	-4.83439	1.95049	-1.74743
H	-6.27792	3.962757	-1.66019
H	-4.17614	-5.31487	1.835799
H	-2.74872	-3.29376	1.761268
H	-4.83413	-1.94731	-1.75158
H	-6.27538	-3.96142	-1.67029
H	4.175427	5.315996	1.834798
H	2.749031	3.294234	1.761958
H	4.833698	1.946875	-1.75087
H	6.275049	3.960971	-1.67048
H	6.282361	-3.96003	-1.65622
H	4.83869	-1.94793	-1.74408
H	2.744084	-3.29338	1.763621
H	4.172798	-5.31319	1.843718
H	-5.95028	5.658329	0.134406

H	-5.94822	-5.66032	0.121153
H	5.946935	5.661339	0.119504
H	5.951145	-5.65792	0.135439

C_{60} E(RBELYP) = -2286.17440820 a.u.

C	-1.45066	-2.59124	-1.94417
C	-2.28223	-2.61376	-0.75198
C	-3.15402	-1.55629	-0.48916
C	-3.2311	-0.43226	-1.40818
C	-2.43289	-0.41096	-2.55228
C	-1.52475	-1.51267	-2.82603
C	-0.15872	-3.16379	-1.60302
C	-0.19124	-3.53954	-0.199
C	-1.50357	-3.19981	0.326867
C	-1.62769	-2.70439	1.625165
C	-3.28342	-1.04051	0.863851
C	-3.40887	0.77853	-0.62315
C	-2.78053	1.961418	-1.01438
C	-1.94908	1.983528	-2.2069
C	-1.77933	0.821737	-2.96034
C	-0.46701	0.481535	-3.48543
C	-0.30987	-0.96124	-3.40329
C	0.930184	-1.51002	-3.07513
C	1.007268	-2.63413	-2.15655
C	0.943526	-3.37005	0.594467
C	2.158669	-2.81823	0.017928
C	2.190052	-2.45801	-1.32944
C	2.84359	-1.22516	-1.73701
C	2.064785	-0.63905	-2.81577
C	1.914105	0.745413	-2.89542
C	0.622172	1.3172	-3.237
C	0.445281	2.528064	-2.45227
C	-0.81379	2.853545	-1.94707
C	-3.44081	0.402737	0.781099
C	0.158684	3.163777	1.603032
C	-1.00724	2.634222	2.156647
C	-0.93013	1.510003	3.075212
C	0.309826	0.961191	3.40329
C	1.52471	1.512618	2.826045
C	2.282162	2.613638	0.751978
C	1.503514	3.199864	-0.32687
C	0.191253	3.539621	0.199046
C	-0.9435	3.370033	-0.59455

C	-2.15876	2.81836	-0.01795
C	-2.18997	2.457975	1.329373
C	-2.06478	0.639076	2.815688
C	-1.91398	-0.74536	2.895172
C	-0.62219	-1.31732	3.237077
C	0.467004	-0.48166	3.485419
C	1.779294	-0.82175	2.96033
C	2.432937	0.410881	2.552345
C	3.231109	0.432232	1.408218
C	3.153987	1.556326	0.48916
C	1.627745	2.704552	-1.62518
C	2.535379	1.602479	-1.89883
C	3.283359	1.040511	-0.86387
C	3.440833	-0.40267	-0.78109
C	3.40883	-0.77846	0.623146
C	2.780668	-1.96141	1.014373
C	1.949161	-1.98349	2.206823
C	0.813746	-2.85344	1.947039
C	-0.44529	-2.52831	2.452458
C	-2.84367	1.225234	1.737026
C	-2.53538	-1.60244	1.898877
C	1.450624	2.591075	1.944172

NR E(RB3LYP) = -1032.92757819 a.u.

C	3.18536	-0.256	-0.03936
C	2.181566	0.736482	0.07085
C	0.845792	0.375626	0.041942
C	0.416954	-0.9597	-0.09997
C	1.419585	-1.94022	-0.21184
C	2.760378	-1.61022	-0.18234
H	2.416297	1.789046	0.158166
H	1.106432	-2.97474	-0.31503
H	3.490468	-2.40617	-0.25023
N	-0.91526	-1.29672	-0.12845
O	-0.08294	1.374834	0.146604
C	-1.41862	1.065163	0.11557
C	-2.31686	2.076315	0.218579
C	-3.75166	1.821149	0.195072
C	-4.17957	0.395908	0.054335
C	-3.2339	-0.64674	-0.0516
C	-1.79905	-0.33931	-0.02607
H	-1.98662	3.104167	0.321572
O	-4.57584	2.733998	0.287332

C	-3.68207	-1.97262	-0.18151
C	-5.54813	0.098931	0.028839
C	-5.04234	-2.25222	-0.20497
C	-5.98061	-1.21529	-0.09993
H	-5.37872	-3.28079	-0.30557
H	-2.94546	-2.76461	-0.26216
H	-7.04372	-1.43974	-0.11952
H	-6.24675	0.925175	0.111904
N	4.522342	0.075472	-0.007
C	5.571749	-0.91303	-0.25743
H	5.234379	-1.61101	-1.03008
H	6.424083	-0.37837	-0.69149
C	4.971698	1.439356	0.27291
H	5.955174	1.366502	0.750647
H	4.309841	1.889588	1.019606
C	6.0247	-1.6716	0.996482
H	5.197689	-2.23291	1.442607
H	6.82484	-2.37781	0.746149
H	6.408163	-0.98037	1.755187
C	5.068776	2.330527	-0.97175
H	5.429	3.328912	-0.69803
H	5.767029	1.907053	-1.70233
H	4.096119	2.439024	-1.46167

DMDP E(RB3LYP) = -1052.31092472 a.u.

C	-3.45683	1.129194	-0.08604
C	-4.39687	0.065727	-0.00383
C	-3.86582	-1.24533	0.098814
C	-2.49846	-1.46327	0.118474
C	-1.56412	-0.41184	0.033278
C	-2.09522	0.891491	-0.07004
H	-3.79773	2.155568	-0.14401
H	-4.52597	-2.10264	0.145948
H	-2.13453	-2.48575	0.194058
N	-5.75552	0.29955	-0.02205
C	-6.72443	-0.77135	0.207914
H	-7.61111	-0.31685	0.664103
H	-6.32588	-1.46379	0.956572
C	-6.3084	1.633841	-0.25401
H	-7.28531	1.502543	-0.73289
C	-0.14761	-0.71297	0.05672
H	0.099838	-1.76886	0.148391
C	0.88587	0.164145	-0.02163
H	0.709652	1.231652	-0.11637

C	2.276261	-0.20501	0.010743
C	3.316886	0.680482	-0.0681
O	2.512682	-1.55057	0.130175
C	4.675527	0.240141	-0.02833
H	3.093047	1.736918	-0.16234
C	3.795171	-2.01671	0.171304
C	4.858531	-1.18582	0.097946
H	5.858487	-1.60187	0.13454
C	5.757026	1.121841	-0.10593
C	5.547434	2.523471	-0.23037
N	5.335343	3.666038	-0.33169
C	7.095549	0.642433	-0.06277
N	8.179338	0.212406	-0.02406
C	3.839042	-3.50365	0.302701
H	3.323848	-3.98019	-0.53975
H	3.332745	-3.8245	1.220869
H	4.872504	-3.85528	0.328564
H	-5.68694	2.160102	-0.98547
H	-1.4262	1.745315	-0.12988
C	-6.46851	2.471553	1.02103
C	-7.13093	-1.52722	-1.06362
H	-7.1301	1.971827	1.737425
H	-6.90357	3.449238	0.783412
H	-5.50476	2.634933	1.51352
H	-7.57873	-0.84734	-1.79695
H	-7.86829	-2.30307	-0.82669
H	-6.26721	-2.00623	-1.53545

PDVB monomer E(RB3LYP) = -387.048584606 a.u.

C	-0.000140495	-0.000103992	-0.000003485
C	0.000086851	0.000014244	0.000013297
C	-0.000011557	-0.000073858	0.000011360
C	0.000058422	0.000045733	0.000004335
C	0.000030203	-0.000084046	0.000023944
C	0.000028144	0.000169799	-0.000031861
C	-0.000064102	0.000011708	-0.000014803
C	0.000033916	0.000065147	-0.000004659
C	-0.000037265	0.000048856	-0.000018858
C	-0.000043883	-0.000057704	0.000001592
H	0.000028134	0.000006619	0.000004673
H	-0.000017216	0.000012184	-0.000005847
H	-0.000018292	0.000009090	-0.000006193

H	0.000007141	-0.000027985	0.000006892
H	0.000021638	-0.000013148	0.000006775
H	-0.000010535	-0.000018558	0.000001024
H	0.000000494	-0.000016962	0.000002830
H	0.000021273	-0.000013143	0.000007165
H	0.000011912	0.000018024	-0.000000273
H	0.000015217	0.000007991	0.000002091
